# NRT1.1 as central signaling hub orchestrating plant growth and stress trade-offs

**DOI:** 10.3389/fpls.2026.1965737

**Published:** 2026-09-23

**Authors:** Ranran Liu, Shulei Wang, Yanting Wang, Jie Song

**Affiliations:** 1 College of Agriculture and Biology, Liaocheng University, Liaocheng, China; 2 College of Life Science, Shandong Normal University, Jinan, China

**Keywords:** ABA receptor, crop improvement, NRT1.1, root architecture, signal integration

## Abstract

Plants must continuously balance nitrogen-dependent growth with adaptation to environmental stresses. The nitrate transporter AtNRT1.1 in *Arabidopsis thaliana* and its rice ortholog OsNRT1.1B have been reported to integrate nitrogen- and stress-related signals in their respective species. This review synthesizes how NRT1.1 orthologs coordinate nitrate and stress signaling. Experimentally characterized NRT1.1 forms a homodimer, and phosphorylation of a key threonine residue (Thr101) acts as a molecular switch, toggling between high- and low-affinity nitrate transport modes. OsNRT1.1B also functions as a high-affinity ABA receptor in rice. NRT1.1 orthologs may integrate nitrate and ABA signals via allosteric regulation and ligand competition. Signaling triggers cellular responses via calcium signatures and kinase cascades, driving transcriptional reprogramming through NLP transcription factors, including AtNLP2/7 in *Arabidopsis* and OsNLP3/4 in rice. These responses include root architecture remodeling, rhizosphere homeostasis, nitrogen-phosphorus coordination, and shoot-root developmental synchronization. This review also discusses the evolution of the NRT1.1 signaling, highlighting experimentally supported core functions and lineage-specific innovations. The nitrate transport/signaling module discussed here is supported primarily by studies of AtNRT1.1 in *Arabidopsis*, whereas the ABA-receptor module is supported mainly by studies of OsNRT1.1B in rice. However, whether mechanisms established in *Arabidopsis* are conserved and agronomically relevant in other crop species remains to be fully established. Although nitrate- and ABA-related NRT1.1 functions have both been investigated in rice, direct experimental evidence that the two proposed modules operate coordinately within a single species remains limited. Accordingly, this model should be viewed as a comparative and hypothesis-generating framework rather than a universally validated mechanism. Understanding this network may provide a basis for identifying targets to improve nutrient use efficiency, stress resilience, and yield stability, pending validation in target crops.

## 1 Introduction

Plants in natural and agricultural ecosystems face a central trade-off: how to allocate limited resources between growth and survival. This challenge is often described as a growth–stress trade-off, in which the activation of developmental programs under favorable conditions must be balanced against the induction of defense mechanisms during abiotic stresses such as drought and salinity (Ma et al., 2025; Wang and Zhu, 2025). To ensure high yield, large quantities of nitrogen fertilizer are often applied in agricultural production; however, excessive nitrogen application can reduce nitrogen use efficiency (NUE), defined here as the proportion of applied nitrogen fertilizer that is recovered and utilized by plants to produce biomass and grain yield. It can also cause severe environmental degradation, including soil acidification and water pollution (Zhang et al., 2013; Wang et al., 2018a). Improving NUE therefore requires not only optimizing nitrogen acquisition and assimilation but also understanding how plants coordinate nitrogen-dependent growth with stress adaptation under fluctuating environmental conditions. Such knowledge may help identify regulatory targets for developing crops that maintain productivity while using nitrogen more efficiently and coping with environmental stresses.

At the core of the regulatory network governing nitrate-dependent growth and stress adaptation lies nitrate (NO_3_^-^), the primary nitrogen source for most plants in aerobic soils. Nitrate is not only a major nutrient but also serves as a critical signaling molecule, the availability of which is highly heterogeneous in space and time (Crawford, 1995; Rashid et al., 2018; Liu et al., 2017). It is important to note, however, that ammonium (NH_4_^+^) represents another major nitrogen source, particularly in flooded ecosystems such as rice paddies where it is the predominant form (Wang et al., 2018b). Nitrogen use efficiency in such environments indeed depends on both nitrate and ammonium acquisition and assimilation. Nevertheless, NRT1.1 family proteins function as nitrate transceptors, combining nitrate transport with nitrate sensing. In *Arabidopsis thaliana*, NRT1.1, also known as CHL1 or NPF6.3, was initially characterized as a dual-affinity nitrate transporter mediating nitrate uptake (Tsay, 1993; Huang et al., 1996; Léran et al., 2013). This review therefore deliberately focuses on nitrate-specific signaling pathways. Notably, as detailed in Section 5.2.1, AtNRT1.1 does exhibit functional crosstalk with ammonium metabolism, including its roles in NH_4_^+^ toxicity responses, Cl^-^ homeostasis under salt stress, and rhizosphere pH modulation (Jian et al., 2018; Liu et al., 2020; Zhu et al., 2019), but these functions are mechanistically linked to its nitrate transport/sensing capacity.

AtNRT1.1 activity is modulated by phosphorylation of the key threonine residue Thr101, enabling efficient nitrate acquisition across a wide range of soil nitrate concentrations (Liu et al., 1999). This functional paradigm is evolutionarily conserved and critical in major crops. For instance, orthologs such as OsNRT1.1B in rice (Hu et al., 2015; Wang et al., 2018b; Zhang et al., 2019a) and ZmNRT1.1B in maize (Cao et al., 2024) are pivotal for nitrate uptake and translocation, directly enhancing NUE and grain yield. These findings have established NRT1.1 orthologs as key mediators of nitrogen acquisition, functioning not only as a transporter but also as a sensor that triggers downstream transcriptional reprogramming (Medici and Krouk, 2014; Alvarez et al., 2018), thus making it a key target for breeding nitrogen-efficient crops.

However, recent discoveries that elevate NRT1.1 orthologs are far more than specialized nitrate transporters. The identification of OsNRT1.1B as a high-affinity receptor for the stress hormone ABA has fundamentally transformed our mechanistic understanding of its function (Ma et al., 2025; Wang and Zhu, 2025). This dual perception places NRT1.1 orthologs at the nexus of nutrient and stress signaling, functioning as a molecular integrator at the plasma membrane. It directly perceives and integrates the competing signals of growth (nitrate) and defense (ABA), thus serving as a central signaling node in the plants’ response to its environment (Gong and Yang, 2026).

Building upon this paradigm shift, this review synthesizes the roles of NRT1.1 orthologs in mediating the growth–stress trade-off. We examine the structural basis of its “transceptor” function, explore the molecular logic underlying its integration of nitrate and ABA signals, and delineate the downstream signaling cascades, from immediate calcium signatures to long-term transcriptional reprogramming. By connecting these molecular events to systemic physiological outputs, such as root architecture plasticity and nutrient homeostasis, we aim to provide a unified conceptual framework for understanding how plants navigate environmental complexity. Finally, we discuss the translational potential of engineering this pivotal hub for developing next-generation crops that are both productive and resilient. A comprehensive glossary of all core gene abbreviations used in this review is provided in **Supplementary Table S1**.

## 2 The molecular and structural basis of NRT1.1 orthologs function

The capacity of NRT1.1 to mediate complex physiological responses originate from its unique nature as a “transceptor”, a protein that couples substrate transport with receptor-mediated signaling. This dual functionality is closely linked to its structure and dynamic regulation. This chapter examines the core principles governing NRT1.1 function: its homodimeric scaffold, dual-affinity transport kinetics, and the pivotal Thr101 phosphorylation switch that modulates its activity. Understanding this molecular foundation is essential for appreciating how NRT1.1 operates as a central node for signal integration.

### 2.1 The dimeric framework: a scaffold for dual functionality

At the core of NRT1.1’s transceptor function is its homodimeric structure, a conserved scaffold in NRT1.1 orthologs that organizes key regulatory and functional residues. Each monomer consists of 12 transmembrane (TM) helices adopting an inward-open conformation typical of the Major Facilitator Superfamily (MFS) (Sun et al., 2014; Parker and Newstead, 2014). In the unphosphorylated state, the two monomers form a stable dimer via hydrophobic interactions, primarily at the TM3-TM6 interface. This conformation underpins the low-affinity transport mode and is allosterically modulated by nitrate binding (Sun et al., 2014). Notably, even in the unphosphorylated state, the two protomers exhibit intrinsic asymmetry in both nitrate binding and tunnel dynamics, a feature that may create a structural environment permissive for Thr101 phosphorylation (Yen et al., 2026).

Within this framework, two residues are paramount. His356, located on TM7, plays a central role in nitrate coordination. Its protonation state is pH-dependent: the protonated from stabilizes nitrate binding via interactions with Arg45, whereas deprotonation facilitates nitrate release toward the cytosol through sequential interactions with Arg45 and Lys164. Mutation of this residue abolishes all transport activity (Sun et al., 2014; Parker and Newstead, 2014; Yen et al., 2026). Thr101 resides in a hydrophobic pocket near the dimer interface and serves as the master regulatory switch. Its phosphorylation status directly governs the protein’s monomer-dimer equilibrium and conformational dynamics, thereby controlling its functional output (Sun et al., 2014). Recent molecular dynamics (MD) simulations further reveal that Thr101 phosphorylation enhances the flexibility of TM2 and TM4, promotes asymmetric movement between the two protomers, and modulates the transition between low and high affinity states (Yen et al., 2026). The synergy among these features provides the structural blueprint for NRT1.1 orthologs’ ability to sense and respond to fluctuating nitrate availability.

### 2.2 The Thr101 phosphorylation switch: a nexus of conformation and function

The transition between these affinity states is governed by a precise molecular switch: the phosphorylation of Thr101. This post-translational modification fundamentally determines the functional state of AtNRT1.1 by remodeling its homodimeric assembly and inter-protomer conformational dynamics. In the unphosphorylated state, which predominates under high-nitrate conditions, AtNRT1.1 exists as a stable dimer, corresponding to the low-affinity transport mode. Upon phosphorylation at Thr101, which is induced by low external nitrate, the dimer interface is destabilized, shifting the protein into its high-affinity conformation (Liu and Tsay, 2003; Parker and Newstead, 2014; Sun et al., 2014).

Molecular dynamics simulations have further illuminated this process. Yen et al. (2026) showed that Thr101 phosphorylation alters the conformational flexibility of TM2 and TM4, thereby modulating the probability of occluded state formation. Meanwhile, Subhadarshini et al. (2025) proposed that phosphorylation reduces the overall conformational transition rate, establishing a rate–affinity trade-off whereby slower conformational changes enhance substrate retention and thus transport efficiency at low nitrate concentrations. Definitive genetic evidence comes from studies of Thr101 mutants. The phosphomimetic AtNRT1.1Thr101D mutant is locked in a high-affinity state, while the non-phosphorylatable AtNRT1.1 Thr101A mutant remains in a low-affinity state; both lose biphasic kinetics, demonstrating that Thr101 phosphorylation is the key switch (Parker and Newstead, 2014). Furthermore, Yen et al. (2026) observed that phosphorylation induces asymmetric separation of TM2 from TM3 in the two subunits, suggesting that the two protomers within the AtNRT1.1 dimer may adopt distinct functional states. Together, these findings establish Thr101 as the regulatory nexus of NRT1.1’s transceptor function, converting a simple chemical modification into a profound shift in both transport efficiency and signaling intensity. The key functional residues of AtNRT1.1 and their mutant phenotypes are summarized in **Table 1**.

**Table 1:** Comparison of function and cross-species conservation of key functional residues in AtNRT1.1 and OsNRT1.1B.

Residue site	Functional category	Function in *A. thaliana* NRT1.1	Status in *O. sativa* NRT1.1B	Conservation level	Reference(s)
Thr101	Basal nitrate transport (dual-affinity switch)	Phosphorylated by AtCIPK23; mediates the transition between high- and low-affinity nitrate transport modes	Corresponding residue is conserved; dual-affinity regulatory function has been validated	Highly conserved (core functional site)	Sun et al., 2014; Parker and Newstead, 2014
His356 (*A. thaliana*)/His362 (*O. sativa*)	Basal nitrate transport (substrate coordination)	Core coordinating residue for proton-dependent nitrate binding; determines substrate binding affinity	Corresponding residue is conserved; substrate coordination function has been validated	Highly conserved (substrate-binding core)	Sun et al., 2014; Parker and Newstead, 2014
Arg45	Basal nitrate transport (substrate binding and release)	Participates in nitrate binding and conformational release; mutation disrupts nitrate binding and impairs transport activity	Corresponding residue is conserved; structural role in substrate coordination is preserved	Highly conserved (structural core)	Sun et al., 2014
Arg48	Stress-responsive regulation (direct ABA perception)	No equivalent functional site identified	Core binding residue for ABA receptor activity; mediates direct ABA modulation of nitrate transport	Lineage-specific (functional site restricted to monocot lineages)	Ma et al., 2025
Ser585	Stress-responsive regulation (phosphorylation-mediated transport repression)	Phosphorylated by AtSnRK2.2/2.3/2.6; phosphorylation attenuates both high- and low-affinity nitrate transport activity	The C-terminal cytoplasmic region shows sequence conservation, and the site lies within a canonical AtSnRK2 recognition motif; site-specific functional validation is absent in rice	Motif-conserved (function verified only in *A. thaliana*)	Su et al., 2021

### 2.3 Dual-affinity transport: a dynamic design for environmental adaptation

A hallmark of NRT1.1 is its kinetic plasticity, manifested as biphasic transport kinetics that enable switching between high- and low-affinity modes. This adaptability is crucial for plants thriving in soils with fluctuating nitrate availability. Under low nitrate conditions (< 1 mM), AtNRT1.1 operates in a high-affinity mode (K_m_ ≈ 40–50 µM) to efficiently acquire nitrate. Conversely, when nitrate is abundant (> 1 mM), it transitions to a low-affinity mode (K_m_ ≈ 4 mM), facilitating bulk uptake while preventing nitrate cytotoxicity (Ho et al., 2009; Parker and Newstead, 2014; Sun et al., 2014). This intrinsic kinetic property has been demonstrated in heterologous systems such as *Xenopus oocytes* (Liu et al., 1999; Ho et al., 2009).

Crucially, this kinetic switch is not merely a transport adjustment; it is intimately linked to NRT1.1 orthologs signaling capacity under different nitrate regimes. It should be clarified that NRT2 family transporters, predominantly AtNRT2.1 and AtNRT2.2, are the major contributors to sustained high-affinity nitrate uptake in *Arabidopsis*. Concomitant disruption of *AtNRT2.1* and *AtNRT2.2* reduces the inducible high-affinity transport system (IHATS) by up to 72-80%, whereas the contribution of AtNRT1.1 to high-affinity uptake is limited to approximately 10% under comparable condition (Li et al., 2007). Moreover, NRT2.1 itself participates in nitrate signal transduction; in *Lotus japonicus*, nitrate transport via LjNRT2.1 is a key step in the NLP (NIN-LIKE PROTEIN) signaling pathway that controls nodulation, and LjNRT2.1-mediated nitrate uptake/transport is required for LjNLP4 nuclear localization and the consequent induction or repression of symbiotic genes (Misawa et al., 2022). In contrast, the high-affinity state of AtNRT1.1 primarily serves as a sensory module that enables rapid adaptive responses under fluctuating nitrate concentrations, rather than mediating bulk high-affinity nitrate uptake (Ho et al., 2009). Because this review focuses on the NRT1.1-centered nitrate transport–sensing framework, it does not aim to provide a comprehensive analysis of NRT2 systems in cereal crops. We acknowledge that NRT2 homologs have undergone extensive lineage-specific duplication in the Triticeae, and that their regulatory mechanisms may therefore differ from those described in *Arabidopsis* (Feng et al., 2025; Nishida et al., 2025; Liu et al., 2026).

Functionally, the low-affinity (dephosphorylated) state of AtNRT1.1 is predominantly associated with robustly triggering the primary nitrate response (PNR), whereas the high-affinity (phosphorylated) state, while also capable of initiating PNR, primarily acts as a sensor enabling rapid adaptive responses under fluctuating nitrate concentrations (Ho et al., 2009; Medici and Krouk, 2014). The low-affinity state, operating under nitrate-replete conditions, drives broader and more sustained transcriptional reprogramming encompassing nitrogen metabolism, root developmental patterning, and nutrient crosstalk (Wang et al., 2020a). Thus, transport through AtNRT1.1 is inherently coupled to perception, synchronizing nutrient influx dynamics with real-time signaling output.

Here we explicitly define the two hierarchical levels of nitrate transcriptional response to eliminate ambiguity:

1. Primary nitrate response (PNR) refers to the rapid (minutes to 1–2 hours), protein synthesis-independent induction of a conserved core set of nitrate-responsive genes upon nitrate exposure. This includes key genes in nitrate assimilation (e.g., *AtNIA1, AtNiR*) and high-affinity transport (e.g., *AtNRT2.1*), which are directly activated by NLP transcription factors. PNR represents the immediate-early, core signaling output of nitrate perception (Medici and Krouk, 2014).
2. Extensive transcriptional reprogramming refers to the sustained, genome-scale transcriptional remodeling occurring at later time points (hours to days). It builds upon the primary response and integrates secondary signals (carbon status, hormone crosstalk, multi-nutrient homeostasis), encompassing hundreds of genes involved in carbon metabolism, root architecture patterning, and stress adaptation. This broader reprogramming requires sustained nitrate signaling and is more prominently associated with the low-affinity state of NRT1.1 under nitrate-replete conditions (Wang et al., 2020a).

## 3 The core regulatory logic of NRT1.1: a proposed two-module framework for the growth–stress trade-off

NRT1.1 orthologs regulate the growth-stress trade-off through a sophisticated two-module system. The first module, governed by the Thr101 phosphorylation switch, senses nitrogen availability to establish a “growth baseline”. The second module, which functions as a high-affinity ABA receptor, acts as a priority switch to override this baseline under stress. This chapter dissects these two modules and then describes their integration into a hierarchical decision-making model. **Figure 1** provides a conceptual overview of the hierarchical regulatory network orchestrated by NRT1.1 orthologs. This framework sets the stage for the following detailed discussion of the two-module system, downstream signaling, and physiological outputs.

**Figure 1:**
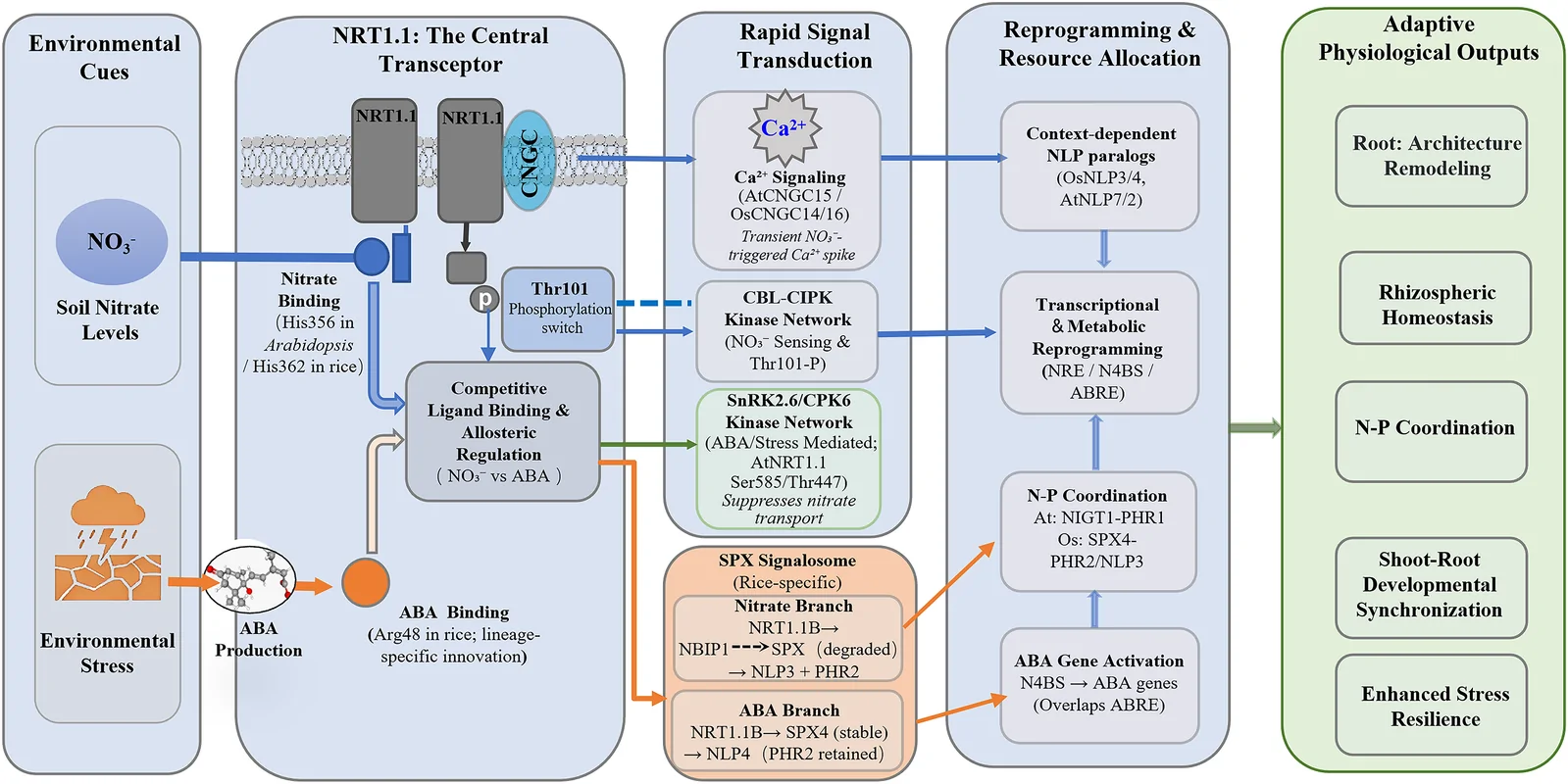
Hierarchical signal integration network orchestrated by NRT1.1 ortholog transceptors in balancing nitrate-dependent growth with abiotic stress tolerance. The diagram summarizes experimentally supported NRT1.1-related signaling branches, with emphasis on *Oryza sativa* OsNRT1.1B and comparative evidence from *Arabidopsis thaliana*. In rice, the nitrate-responsive branch involves OsNRT1.1B–OsSPX4-dependent regulation of OsNLP3/OsPHR2, whereas the ABA-responsive branch involves ABA binding to OsNRT1.1B and modulation of the OsNRT1.1B–OsSPX4–OsNLP4 regulatory module. These branches have been characterized under different nitrate regimes and experimental timescales and should therefore not be interpreted as a single, fully validated linear pathway. Solid arrows indicate experimentally supported relationships within the corresponding species and experimental context. Dashed arrows indicate proposed crosstalk, indirect relationships, extrapolations from comparative evidence, or mechanisms that remain unresolved. Sequence conservation of NRT1.1-related residues does not, by itself, establish functional conservation among species. The Thr101-dependent phosphorylation switch is established for *Arabidopsis* NRT1.1, whereas its functional equivalence in rice NRT1.1B remains to be determined. The proposed coordination between nitrate-responsive and ABA-responsive branches, including their temporal relationship and possible competition for SPX4-dependent regulation, requires further experimental validation. NLP activity is modulated by multiple nitrate-sensing pathways, including NRT1.1-dependent and -independent routes (e.g., autonomous intracellular nitrate sensing by AtNLP7). Unresolved mechanistic gaps distinguishing CNGC-dependent transient Ca²^+^ spikes (AtNRT1.1-AtCNGC15 in *Arabidopsis*; OsNRT1.1B-OsCNGC14/16 in rice) from sustained low-nitrate calcium signatures controlling long-term NLP nuclear retention are discussed in the main text. Line and color coding rules: Grey, sequence-conserved or generally characterized NRT1.1 features; functional equivalence across species is not implied.; orange, rice-specific ABA/SPX4/NLP4 module (absent in *Arabidopsis*); green, *Arabidopsis*-specific SnRK2/CPK6 phosphorylation of AtNRT1.1 at Ser585 to suppress nitrate transport; blue, nitrate-triggered growth cascades. Dashed lines denote unresolved gaps (see main text). ABA, abscisic acid; CBL, calcineurin B-like protein; CIPK, CBL-interacting protein kinase; CNGC, cyclic nucleotide-gated channel; N4BS, NLP4-binding ABA-responsive cis-element; NLP, NIN-like protein; PHR, phosphate starvation response transcription factor; SPX, SYG1/Pho81/XPR1 domain repressor; SnRK2, sucrose non-fermenting 1-related protein kinase 2.

### 3.1 Module I: the Thr101 phosphorylation switch—setting the growth baseline via nitrogen sensing

The phosphorylation state of Thr101 functions as a dynamic switch that translates external nitrogen availability into a cellular “growth baseline”, a process mediated by the AtCBL-AtCIPK kinase network and tightly coupled to auxin signaling to shape plant development.

#### 3.1.1 Dynamic regulation by the CBL-CIPK kinase network

The phosphorylation equilibrium of Thr101 is precisely governed by a conserved AtCBL-AtCIPK signaling cassette (Ho et al., 2009). Under low-nitrate conditions, AtNRT1.1 perceives nitrate scarcity and, via interaction with cyclic nucleotide-gated channels (CNGCs), triggers a specific cytosolic Ca²^+^ signature (Wang et al., 2021). This Ca²^+^ signal activates the AtCBL1/9-AtCIPK23 complex, which phosphorylates Thr101, switching NRT1.1 to its high-affinity mode to prioritize nitrate scavenging (Ho et al., 2009; Wang et al., 2021). Conversely, under high-nitrate conditions, a negative feedback loop involving AtCIPK8 competitively inhibits the AtCBL1/9-AtCIPK23 complex, thereby maintaining Thr101 in its dephosphorylated, low-affinity state to prevent excessive uptake and maintain metabolic homeostasis (Rashid et al., 2020; Subhadarshini et al., 2025).

This phosphorylation event extends beyond transport regulation. For instance, the phosphorylation status directly influences stomatal movement; the phosphomimetic AtNRT1.1Thr101D mutant promotes stomatal closure by inhibiting nitrate-dependent K^+^ influx into guard cells, thereby enhancing drought tolerance (Kou et al., 2025). This establishes the Thr101 switch as a direct link between nitrate sensing and stress adaptation. Furthermore, this regulatory node serves as a platform for integrating other stress signals. The ABA signaling kinase AtSnRK2.6/AtOST1 and the calcium-dependent kinase AtCPK6 phosphorylate AtNRT1.1 at distinct sites (Ser585 and Thr447, respectively) to inhibit nitrate transport during drought, creating key molecular interfaces for signal crosstalk (Su et al., 2021; Ma et al., 2023). In addition, the ABI2–NPF6.3/NRT1.1 regulatory module has been reported to connect ABA signaling with the regulation of nitrate transport and nitrate-dependent growth (Léran et al., 2015). Collectively, these modifications exemplify how diverse stress pathways can directly fine-tune the core nitrate-sensing switch.

#### 3.1.2 Coupling with auxin signaling to coordinate root development

The Thr101 switch in NRT1.1 serves as a critical interface that connects nitrate signaling to developmental programs, particularly auxin-mediated remodeling of root architecture. Under nitrogen deficiency, the phosphorylated form of AtNRT1.1 modulates the auxin efflux carrier AtPIN2 to enhance basipetal auxin flux from lateral root primordia (LRP), thereby suppressing their initiation and outgrowth. Concurrently, it downregulates the auxin biosynthesis gene *AtTAR2* in the root stele and the influx carrier *AtLAX3* in overlying tissues of LRP, reducing local auxin accumulation (Bouguyon et al., 2016; Zhang et al., 2019b; Chai et al., 2020; Maghiaoui et al., 2020; Vega et al., 2021). This coordinated action shifts resources toward primary root elongation, a conservative foraging strategy that explores deeper soil layers for nitrogen.

By contrast, under high nitrate, the dephosphorylated form of AtNRT1.1 relieves this repression, enabling localized auxin. This promotes downstream auxin signaling and increases lateral root density and length (e.g., through induction of *AtAFB3* expression) to exploit nutrient-rich patches (Vidal et al., 2010; Maghiaoui et al., 2020). Collectively, this regulatory cascade links the phosphorylation status of AtNRT1.1 to the spatial distribution of auxin and, ultimately, to root system architecture, enabling NRT1.1 to translate a nutrient signal into an adaptive growth phenotype.

### 3.2 Module II: the ABA receptor function—mediating signal priority under stress

Recent studies on the rice ortholog OsNRT1.1B have revealed that NRT1.1 family proteins function as plasma membrane-localized receptors that directly perceive the stress hormone ABA (Ma et al., 2025). This discovery reveals the molecular basis for prioritizing stress responses over growth, establishing NRT1.1 as a safety switch in plant survival.

#### 3.2.1 Molecular mechanism: competitive binding of ABA and Nitrate

Biochemical and structural analyses, including Drug Affinity Responsive Target Stability (DARTS) and Microscale Thermophoresis (MST), reveal that OsNRT1.1B binds ABA with high affinity (Kd ≈ 200 nM), within its physiological range of ABA, and with one order of magnitude stronger than for nitrate (Kd ≈ 200 µM) (Ma et al., 2025; Wang and Zhu, 2025). This ligand selectivity is structurally determined by two spatially adjacent residues: Arg48 exclusively mediates ABA recognition, whereas the conserved His362 coordinates nitrate ions. The overlapping binding pocket enables reciprocal ligand displacement: elevated ABA displaces nitrate to activate stress programs, while high nitrate concentrations attenuate ABA signal output (Gong and Yang, 2026; Ma et al., 2025). OsNRT1.1B therefore functions as an extracellular ABA/nitrate ratio biosensor to set growth-stress signal priority.

Ligand competition is supplemented by rapid post-translational inhibitory regulation under stress. Activated SnRK2 kinases such as SnRK2.6/OST1 directly phosphorylate AtNRT1.1 at Ser585 to suppress dual-affinity nitrate transport capacity (Su et al., 2021). This kinase-dependent inhibition operates independent of ligand displacement, forming a dual-layer regulatory system that rapidly suppresses nutrient uptake upon stress onset.

From an evolutionary perspective, functional residues display divergent conservation patterns across terrestrial plant NRT1.1 orthologs. Basal nitrate transport determinants, the Thr101 phosphorylation switch and the His356/His362 nitrate-coordinating site, are fully conserved in all tested lineages (Parker and Newstead, 2014; Sun et al., 2014). The SnRK2 phosphorylation motif surrounding AtNRT1.1 Ser585 shows broad sequence conservation across species, yet its functional repressive effect is only validated in *Arabidopsis*, lacking cross-species genetic evidence. By contrast, the rice-specific Arg48 ABA-binding pocket has no functional equivalent site in AtNRT1.1, demonstrating that direct ABA sensing via NRT1.1 is a monocot-specific evolutionary innovation, while SnRK2-mediated indirect ABA repression represents a widely conserved regulatory module balancing growth and stress. A full summary of key residues and conservation status is provided in **Table 1**.

#### 3.2.2 Physiological integration: stress signal override and resource reallocation

Functioning as a dual receptor, OsNRT1.1B serves as a central hub that allocates resources via competitive ligand binding. Under high-nitrate conditions, nitrate binding promotes the recruitment of the E3 ligase OsNBIP1, leading to ubiquitination and degradation of the repressor SPX4 (SPX domain protein 4). This releases the OsSPX4-sequestered transcription factors OsNLP3 and OsPHR2 (PHOSPHATE STARVATION RESPONSE 1), activating programs for nitrogen/phosphorus utilization and growth (Hu et al., 2019; Wang et al., 2025).

While OsNLP4 is canonically recognized as a nitrate-responsive transcription factor that binds nitrate response elements (NRE) to activate nitrogen assimilation genes (Wu et al., 2021), its capacity to also mediate ABA-dependent stress responses raises a mechanistic question (Wang and Tsay, 2025). Two lines of evidence resolve this dual functionality. First, at the promoter level, OsNLP4 exhibits dual cis-element specificity: it recognizes canonical NRE motifs in nitrate metabolic genes, whilst exclusively targeting the distinct N4BS motif (core CACGTCGC) to induce ABA-responsive stress genes (Ma et al., 2025). Second, at the receptor level, signal priority is established by ligand competition at the OsNRT1.1B pocket. The two transcriptional programs (NRE- vs. N4BS-driven) operate independently downstream, but hierarchical crosstalk occurs upstream at the OsNRT1.1B hub: nitrate saturation degrades OsSPX4 to release both OsNLP3 and OsNLP4 for growth programs, whereas elevated ABA outcompetes nitrate, stabilizing OsSPX4 to selectively liberate OsNLP4 for initiating drought defense.

In essence, the ABA receptor function of OsNRT1.1B acts as a key “safety switch.” Through differential ligand affinity, it ensures that urgent stress signals (ABA) override favorable growth signals (nitrate), enabling plants to make optimal survival decisions amidst conflicting environmental cues.

### 3.3 The logic of signal synergy and integration: a hierarchical decision-making model

NRT1.1 orthologs function as a central signaling hub, integrating nitrogen and stress signals rather than processing them through separate or additive pathways. This integration is achieved through the synergy of its dual functions as a phosphorylation switch and an ABA receptor. This synergy establishes a hierarchical decision-making framework, termed the “basal setpoint and priority override” model, that dynamically optimizes resource allocation between plant growth and stress responses.

#### 3.3.1 Cross-species conservation of core functional modules and the rationale for integrative modeling

Before integrating the regulatory mechanisms characterized in *Arabidopsis* NRT1.1 and *O. sativa* OsNRT1.1B, we first clarify the genomic orthology context to establish the scope of cross-species comparison. *Arabidopsis* harbors a single-copy *NRT1.1* gene that fulfills all nitrate transport and sensing functions. In contrast, rice possesses three paralogs (*OsNRT1.1A/B/C*) with clear subfunctionalization: OsNRT1.1B localizes to the plasma membrane and serves as the direct functional ortholog of AtNRT1.1, whereas OsNRT1.1A mediates vacuolar nitrate storage and OsNRT1.1C exhibits restricted tissue expression (Hu et al., 2015; Wang et al., 2018b). All cross-species comparisons in this review are therefore strictly limited to OsNRT1.1B.

Building on the structural and mechanistic details established in Sections 2.1, 2.2, 3.1.1 and 3.2.1, cross-species comparisons reveal a layered evolutionary pattern: the core nitrate transport machinery (centered on His356/His362 coordination and Arg45 electrostatic relay) and Thr101 phosphorylation switch are sequence-conserved across all examined angiosperms, with the mechanistic models of the phosphorylation switch extensively validated in *Arabidopsis* (Sun et al., 2014; Parker and Newstead, 2014; Yen et al., 2026; Subhadarshini et al., 2025); the upstream AtCBL-AtCIPK regulatory backbone shows broad sequence conservation, though direct phosphorylation of OsNRT1.1B by rice CIPK homologs remains experimentally unconfirmed (Ho et al., 2009; Subhadarshini et al., 2025). In sharp contrast, high-affinity ABA perception via Arg48 is a rice-specific lineage innovation, with no equivalent functional site present in AtNRT1.1 (Ma et al., 2025; Gong and Yang, 2026). This residue-level divergence sets a definitive boundary for cross-species generalization.

This layered evolutionary architecture provides a rigorous three-tiered rationale for constructing an integrative conceptual framework, rather than asserting a universally identical pathway. Evolutionarily, the conserved core modules, dual-affinity transceptor function, Thr101 phosphorylation switch, and downstream NLP-dependent transcriptional cascade, likely predate dicot-monocot divergence, constituting a putatively ancestral regulatory toolkit for nitrogen signal balancing in flowering plants. Methodologically, the *Arabidopsis* system delivers atomic-resolution structural dissection and high-precision genetic validation of core transport and phosphorylation mechanisms, while the rice system complements this with agronomically relevant stress phenotyping and novel hormone-sensing discoveries, together forming a multi-scale evidence chain unavailable from any single species. Conceptually, the integrated model generalizes only the shared core decision-making logic of the NRT1.1 hub, basal nitrogen homeostasis modulated by stress priority override, and explicitly attributes all lineage-specific components to their respective species throughout the text to prevent unfounded cross-species extrapolation.

#### 3.3.2 Core integration mechanism: interplay of phosphorylation-dependent kinetics and ligand-directed signaling

The molecular basis for NRT1.1 orthologs signal integration lies in the dynamic interplay between its phosphorylation-dependent transport kinetics and ligand-directed signaling pathway selection. Together, these mechanisms form the foundation for mediating trade-off decisions.

The phosphorylation state of the Thr101 residue in AtNRT1.1, which reflects cellular nitrogen status, dictates the transporter’s kinetic properties. Dephosphorylation of Thr101 in AtNRT1.1 under high-nitrate conditions induces a conformational state that supports high-capacity, low-affinity nitrate transport, thereby channeling resources towards growth-promoting programs (Parker and Newstead, 2014). Conversely, phosphorylation under low-nitrate conditions stabilizes a high-affinity, low-capacity conformation. This not only facilitates the uptake of scarce nitrate but also primes the system for energy-conserving stress responses (Fang et al., 2021; Subhadarshini et al., 2025; Yen et al., 2026).

Concurrent with this kinetic regulation, competition between nitrate and ABA for the overlapping binding pocket on NRT1 family proteins determines distinct downstream signaling outputs in different plant species. In rice, elevated ABA levels competitively displace nitrate from OsNRT1.1B via the ABA-specific binding residue Arg48. This binding event stabilizes the interaction between OsNRT1.1B and the cytoplasmic repressor OsSPX4, leading to the release of the transcription factor OsNLP4 from the SPX4 complex. This cascade subsequently initiates rice stress-responsive pathways, including stomatal closure and osmolyte synthesis (Ma et al., 2025; Wang and Zhu, 2025). In *Arabidopsis*, a separate and parallel regulatory mechanism operates: stress-activated SnRK2 kinases (AtSnRK2.2/2.3/2.6) directly phosphorylate AtNRT1.1 at the Ser585 residue. This phosphorylation attenuates both low- and high-affinity nitrate transport activities of AtNRT1.1, forming a rapid positive feedback loop that prioritizes defense over growth (Su et al., 2021). Thus, phosphorylation-dependent transport regulation and ligand-directed signaling are not parallel inputs within a single universal pathway, but rather constitute a dynamically coupled regulatory circuit that is implemented through distinct, lineage-specific molecular components in rice and *Arabidopsis*.

#### 3.3.3 The “basal setpoint” and “priority override” decision-making model

This integrated molecular logic gives rise to the “basal setpoint and priority override” decision-making model, which translates multidimensional signals into graded and directional physiological outputs to regulate resource allocation.

The “basal setpoint” is established by the nitrogen-sensing AtCBL-AtCIPK network, which translates ambient nitrate levels into the phosphorylation state of Thr101. This sets a baseline for resource allocation: a conservative, energy-saving mode under nitrogen limitation versus a proactive, growth-oriented mode under nitrogen sufficiency (Subhadarshini et al., 2025; Yen et al., 2026).

The “priority override” mechanism, through which stress signals override the nitrogen-defined basal setpoint, is implemented through distinct molecular components in different plant lineages. In rice, when ABA concentrations surpass a physiological threshold, ABA competitively binds to OsNRT1.1B, stabilizing the OsNRT1.1B–OsSPX4 interaction and selectively releasing the transcription factor OsNLP4. OsNLP4 then activates rice-specific stress-responsive genes such as *OsLEA3* via direct binding to the N4BS *cis*-element (core CACGTCGC) in their promoters, while simultaneously suppressing growth-promoting pathways (Ma et al., 2025; Wang and Zhu, 2025). In *Arabidopsis*, the activation of stress-responsive genes like *AtRD29A* is mediated by the canonical PYL–PP2C–SnRK2–ABF signaling cascade and does not involve AtNLP4, which functions primarily in nitrate signaling (Liu et al., 2017; Wang and Zhu, 2025). Therefore, the OsNRT1.1B–OsSPX4–OsNLP4 module and the *Arabidopsis* SnRK2-mediated pathway represent two independent, lineage-specific regulatory mechanisms that should not be generalized as a unified cross-species cascade.

Importantly, this system allows for a graded, rather than binary, response. Under intermediate conditions of moderate stress or nitrogen availability, the relative concentrations and affinities of nitrate and ABA are integrated at the NRT1.1 orthologs hub, resulting in a finely tuned allocation of resources between growth and defense. In essence, NRT1.1 acts as both a nitrogen rheostat setting the baseline for growth and a stress arbiter ensuring survival. Crucially, this molecular model is supported by physiological and agronomic evidence. Field trials demonstrated that under combined low nitrogen and drought stress, rice *nrt1.1b* mutants exhibited significantly greater yield loss than wild-type plants. This difference was substantially diminished under high nitrogen and drought conditions, indicating that nitrogen status modulates the plant’s capacity to respond to stress (Ma et al., 2025). These findings confirm that NRT1.1 orthologs orchestrate hierarchical, non-additive integration of nitrogen and stress signals, ensuring that survival threats receive the highest response priority.

#### 3.3.4 Convergence and fine-tuning by multidimensional upstream signals

The NRT1.1 ortholog decision-making hub is not isolated; its precision is refined by a multi-layered upstream regulatory network that integrates a broad array of environmental cues. This network combines long-term transcriptional adjustments with immediate physicochemical modulation, creating a “long-term calibration and rapid response” system.

A sophisticated network of transcription factors translates long-term environmental signals into precise regulation of *NRT1.1* expression. In *Arabidopsis*, the GARP-type AtNIGT1 (NITRATE-INDUCIBLE GARP-type transcriptional repressor) subfamily repressors act as core nodes of nitrogen-phosphorus (N-P) crosstalk; under phosphate starvation, AtNIGT1 proteins are induced and directly repress At*NRT1.1* transcription to rebalance nutrient allocation (Maeda et al., 2018; Ueda et al., 2020). Similarly, stress-responsive transcription factors directly target the *NRT1.1* promoter. The AtSTOP1-AtTCP20 (STOP1: sensitive to proton toxicity 1; TCP20: teosinte Branched1/Cycloidea/PCF 20), directly activates *AtNRT1.1* transcription to optimize root-zone pH (Tokizawa et al., 2023). More recently, the BRM–STOP1 pathway was reported to regulate NRT1.1 transcription under acidic conditions, adding a chromatin-level component to the environmental regulation of NRT1.1 (Ye et al., 2026). In rice, OsSNAC1 directly activates *OsNRT1.1A/B*, coupling nitrogen uptake with salt stress tolerance (Qi et al., 2023). Subspecies-specific adaptations also exist; for instance, a positive feedback loop between *OsNR2* (*Oryza sativa* Nitrate Reductase 2, also known as OsNIA2, encoding a high-activity NADH/NADPH-dependent nitrate reductase that catalyzes the rate-limiting step of nitrate assimilation) and OsNRT1.1B enhances rice NUE (Gao et al., 2019), while natural allelic variation in OsTCP19 (a tiller-regulating TCP TF) modulates tillering under low nitrogen (Liu et al., 2021). In addition, rice OsNLP4, nitrate signal master transcription factor) acts downstream of OsNRT1.1B to globally orchestrate nitrate absorption and assimilation gene expression, and natural SNPs within its GAF domain create *indica*-*japonica* divergence in nitrogen utilization capacity (Wu et al., 2021, 2025).

Beyond transcriptional control, NRT1.1 function is directly modulated by local physicochemical signals. At the physical level, the proton-motive force generated by AtNRT1.1-mediated H^+^-NO_3_^-^ symport can energize the secondary transport of other nutrients such as K^+^, coupling nitrate signaling with ion homeostasis (Kou et al., 2025). Furthermore, extracellular pH directly alters the protonation state of key residues (e.g., His356 in AtNRT1.1), which modulates its nitrate binding affinity and transport efficiency, enabling the protein to perceive and adapt to root-zone acidity (Bouguyon et al., 2015).

Thus, this hierarchical upstream network complements the rapid post-translational and ligand-based mechanisms. While the “phosphorylation switch” and “ABA receptor” functions enable immediate and dynamic responses to fluctuating nitrogen and stress levels, the transcriptional and physicochemical inputs establish a calibrated baseline for AtNRT1.1 activity. This multi-tiered regulatory architecture ensures that AtNRT1.1 can robustly integrate diverse environmental information to orchestrate optimal growth-defense trade-offs in complex and changing environments.

In summary, Module I and Module II were originally characterized in different experimental systems, with Module I supported primarily by studies of AtNRT1.1 in *Arabidopsis* and Module II supported mainly by studies of OsNRT1.1B in rice. Although nitrate- and ABA-related NRT1.1 functions have both been investigated in rice, direct experimental evidence that the two modules operate coordinately as an integrated system within a single species and under combined environmental cues remains limited. Therefore, the two-module framework presented here should be regarded as a comparative and hypothesis-generating model rather than a universally validated mechanism. The presence of NRT1.1-related orthologs in multiple plant genomes suggests possible evolutionary conservation but does not by itself establish conservation of their molecular functions.

## 4 Downstream signal transduction and cascade activation by NRT1.1

As a central integrator of nitrogen and stress signals, the transceptor NRT1.1 orthologs orchestrate adaptive cellular and physiological responses. This process is governed by two principal mechanisms: a phosphorylation switch modulated by nitrate availability and competitive binding with ABA. These regulatory inputs initiate a downstream signaling cascade that dynamically allocates resources between plant growth and stress defense. Through the coordination of calcium signals, protein kinase cascades, and transcription factor regulation, NRT1.1 orthologs ultimately fine-tune plant metabolism and NUE, thereby optimizing environmental adaptation.

### 4.1 Activation of rapid signaling networks: second messenger and kinase-mediated responses

Within seconds to minutes of stimulation, a rapid signaling cascade translates the conformational and phosphorylation status of NRT1.1 orthologs into immediate cellular events. This initial phase, driven by the activation of second messengers and kinase networks, lays the groundwork for subsequent long-term transcriptional reprogramming (Riveras et al., 2015; Liu et al., 2017). Central to this process is the generation of distinct Ca²^+^ signatures, whose characteristics are determined by the functional state of AtNRT1.1. These unique Ca²^+^ signatures recruit and activate specific kinase subfamilies, bifurcating the signal into antagonistic pathways that either promote growth or mediate stress defense. This bifurcation enables a dynamic balancing of resource allocation in response to environmental fluctuations.

#### 4.1.1 Differential calcium signaling: NRT1.1 functional state-dependent signal encoding

The second messenger Ca²^+^ functions as a versatile signal whose spatiotemporal characteristics (amplitude, frequency, and duration) are precisely encoded by the functional state of NRT1.1 ortholog. By differentially interacting with downstream effectors, NRT1.1 orthologs generate two distinct Ca²^+^ signatures: a “growth-promoting” signature under nitrogen-sufficient conditions and a “stress-responsive” signature under nitrogen deficiency or stress. This initial signal bifurcation dictates the specificity of downstream physiological outcomes.

In *Arabidopsis*, plasma membrane-localized AtNRT1.1 physically interacts with AtCNGC15 to form a concentration-dependent transceptor-channel complex (Wang et al., 2021). Under low-nitrate conditions, Thr101-phosphorylated AtNRT1.1 represses AtCNGC15 channel activity to restrict Ca²^+^ influx. When external nitrate concentrations rise rapidly, AtNRT1.1 undergoes dephosphorylation, triggering complex dissociation and activating AtCNGC15 to produce a transient (~10 s) cytosolic Ca²^+^ spike specific to acute nitrate resupply events (Wang et al., 2021). In rice, a functionally analogous signaling module operates via the OsNRT1.1B–OsCNGC14/16 complex; conserved His362 on OsNRT1.1B (homologous to AtNRT1.1 His356) mediates nitrate-triggered Ca²^+^ permeation through OsCNGC channels (Wang et al., 2025).

It is essential to strictly separate this short-lived CNGC-dependent Ca²^+^ transient from the sustained, minutes-long intracellular Ca²^+^ influx required for persistent AtNLP7 nuclear retention and long-term nitrate transcriptional reprogramming (Liu et al., 2017). Genetic and pharmacological evidence demonstrates that AtCPK10/30/32-mediated phosphorylation of AtNLP7 at Ser205 depends entirely on sustained low-nitrate calcium signatures, rather than the ~10 s transient spike derived from AtCNGC15 (Liu et al., 2017). Mutant analyses of *chl1* and *cngc15* single/double lines further confirm that sustained Ca²^+^ signals governing NLP activity cannot be fully eliminated when the AtNRT1.1–AtCNGC15 complex is disrupted, indicating the existence of uncharacterized, AtNRT1.1-independent calcium sources for long-term nutrient signaling (Yanagisawa, 2026). As summarized by Yanagisawa (2026), this mechanistic divergence between the CNGC–dependent short–lived Ca²^+^ transient and the minutes–long sustained calcium signal required for AtNLP7 nuclear retention remains unresolved, with mutant evidence pointing to AtNRT1.1–independent intracellular calcium pools that support persistent NLP transcriptional activity under chronic nitrate limitation.

Accordingly, the AtNRT1.1–AtCNGC15 complex acts solely as an acute nitrate resupply sensor, and it does not represent the exclusive upstream source of calcium signals driving chronic low-nitrate NLP activation. Beyond NLP regulation, the nitrate-triggered Ca²^+^ cascade also engages the AtCBL9–AtCIPK23 kinase module to reinforce Thr101 phosphorylation of AtNRT1.1, establishing an incoherent feedforward circuit that balances high-affinity nitrate transport and signal output under fluctuating nitrogen availability (Rashid et al., 2020). Mechanistic gaps between the two distinct calcium signatures are elaborated in the **Figure 1** legend for visual reference.

In contrast, under low-nitrate conditions (where AtNRT1.1 is phosphorylated at Thr101) or upon ABA binding, the signaling pathway shifts. AtNRT1.1 primarily activates a phospholipase C (PLC)-mediated pathway, leading to the generation of inositol trisphosphate (IP_3_) and a subsequent rapid, high-amplitude Ca²^+^ burst from internal stores (Riveras et al., 2015; Wang and Zhu, 2025). This distinct, transient Ca²^+^ signature specifically recruits and activates stress-responsive kinases, such as AtCPK6 and AtSnRK2s. These kinases then rapidly initiate defensive mechanisms, including reactive oxygen species (ROS) scavenging and maintenance of ion homeostasis, enabling immediate decoding and response to stress signals (Ma et al., 2023; Su et al., 2021; Wang and Tsay, 2025).

#### 4.1.2 Selective activation and feedback regulation of kinase networks: bifurcating the signal for growth-stress prioritization

The selective activation of downstream kinase networks is critical for transducing NRT1.1 ortholog-derived signals, directing them into either growth-promoting or stress-responsive pathways. This signaling hub enables plants to prioritize resource allocation between nutrient acquisition and environmental adaptation. It comprises two main branches: growth-promoting kinases (e.g., AtCIPK23, AtCPK10/30/32) and stress-responsive kinases (e.g., AtSnRK2s, AtCPK6). The selective engagement of these kinases underscores the central role of NRT1.1 as a primary signal integration platform (Sun et al., 2014; Su et al., 2021; Wang et al., 2025; Zhao et al., 2025).

Once the nitrate signal is transduced via the AtCBL-AtCIPK phosphorylation cycle (Section 3.1.1), the signal bifurcates into two functionally antagonistic kinase branches. In the growth-promoting branch, nitrate-mobilized Ca²^+^ signatures rapidly activate AtCPK10/30/32, which directly phosphorylate AtNLP7 at Ser205 to promote nuclear retention and activate primary nitrate response genes (Liu et al., 2017). Meanwhile, the stress-responsive branch engages AtSnRK2.6/AtOST1 and AtCPK6: in addition to suppressing nitrate transport through direct phosphorylation of AtNRT1.1 at Ser585 and Thr447 (Section 3.1.1), these kinases target downstream effectors including the anion channel AtSLAC1 to trigger rapid stomatal closure, and ROS scavenging systems to mitigate cellular damage (Su et al., 2021; Ma et al., 2023). In rice, a distinct mechanism involves ABA-bound OsNRT1.1B enhancing its interaction with OsSPX4, which releases the transcription factor OsNLP4 to activate ABA-responsive genes. This pathway synergizes with the canonical PYL-PP2C-SnRK2 pathway to strengthen a rapid defense response (Gong and Yang, 2026; Ma et al., 2025).

Multilayered negative feedback loops are crucial for maintaining signaling fidelity and preventing over-amplification of these antagonistic pathways. Under nitrogen-sufficient conditions, *AtCIPK8* attenuates the growth-promoting signal by competitively binding to AtCBL1/9, thereby blocking the formation of the active AtCIPK23-CBL complex. Simultaneously, protein phosphatases type 2C (PP2Cs), such as AtABI2, dephosphorylate NRT1.1 to maintain its functional homeostasis and prevent excessive nitrogen accumulation (Su et al., 2021; Subhadarshini et al., 2025). In the stress pathway, *AtSnRK2s* activity is tightly controlled by dual negative regulation involving PP2C-mediated dephosphorylation and ubiquitin-proteasome system (UPS)-dependent degradation. This dual control prevents an excessive stress response that would otherwise severely inhibit plant growth (Su et al., 2021).

In summary, AtNRT1.1 functions as a central molecular switch that integrates its own functional status with a sophisticated network of kinases and phosphatases, thereby mediates the rapid and dynamic allocation of signaling resources, fundamentally determining the plant’s ability to balance growth with stress resilience.

### 4.2 Transcriptional control and metabolic reprogramming: shaping long-term phenotypic adaptation

The transient signals initiated by the rapid kinase cascades are ultimately translated into durable phenotypic adaptations through comprehensive transcriptional and metabolic reprogramming. By modulating the activity of key transcription factor networks and coordinating nutrient crosstalk, NRT1.1 mediates this long-term response, providing crucial support for plant growth, development, and stress resilience (Sámano et al., 2024).

#### 4.2.1 The central transcriptional hub: NLP-mediated reprogramming of growth and stress responses

The subtype-specific activation and nucleocytoplasmic trafficking of distinct NLP paralogs can be modulated by NRT1.1 ortholog-derived nitrate and ABA signals, while multiple NRT1.1-independent nitrate perception pathways also trigger NLP activation (Liu et al., 2017; Hu et al., 2019; Ma et al., 2025). In *Arabidopsis*, nitrate-triggered Ca²^+^ signaling, acting through AtCPK10/30/32, governs AtNLP7 nuclear retention; pharmacological and genetic evidence places this cascade downstream of NRT1.1 nitrate perception (Liu et al., 2017). In rice, competitive nitrate-ABA ligand binding at OsNRT1.1B governs differential SPX4 sequestration and nucleocytoplasmic shuttling of OsNLP3 and OsNLP4 (biochemical details in Section 3.2.2). Both paralogs act as core transactivators of nitrate assimilation genes to sustain plant growth and nitrogen use efficiency (Wu et al., 2021, 2025): OsNLP3 mediates nitrate-triggered Ca²^+^ signaling to coordinate basal vegetative development (Wang et al., 2025), while ABA-bound OsNRT1.1B stabilizes OsSPX4 to retain OsNLP3 in the cytoplasm and selectively liberate OsNLP4 for abiotic stress transcriptional reprogramming (Hu et al., 2019; Ma et al., 2025).

Under high-nitrate conditions, dephosphorylated AtNRT1.1 mediates a conserved derepression mechanism that coordinates nitrogen and phosphorus signaling. By facilitating E3 ligase OsNBIP1-dependent degradation of the repressor OsSPX4, AtNRT1.1 concurrently releases NLP transcription factors and the phosphorus signaling master regulator OsPHR2, which co-activate genes for nitrogen and phosphorus uptake and assimilation to support vegetative growth (Hu et al., 2019). Full mechanistic details of this N–P co-regulation pathway and its species-specific divergence are provided in Section 5.2.3.

Under low nitrogen, the AtCIPK23-AtCBL1/9 complex phosphorylates NRT1.1 at the conserved Thr101 residue, switching it to a high-affinity transport state to maximize nitrate acquisition. This phosphorylation event also triggers Ca²^+^ influx via CNGC channels, which reinforces the phosphorylation of AtNLP7/OsNLP3 at their conserved serine sites. This modification enhances their nuclear retention and transcriptional activity, thereby sustaining the expression of nitrogen-responsive genes to maintain nitrogen homeostasis under nutrient-limiting conditions (Liu et al., 2017; Wang et al., 2025). Additionally, NRT1.1 signaling activates other NLP members such as *AtNLP2*, an early nitrate-responsive factor that links nitrogen assimilation to carbon metabolism by upregulating key enzymes in glycolysis and the tricarboxylic acid TCA cycle, thus supplying the carbon skeletons and energy required for nitrogen utilization (Konishi et al., 2021).

Under environmental stress, NRT1.1 utilizes ligand-competitive binding and conformational specificity to divert signaling from growth to defense. In rice, OsNRT1.1B functions as a high-affinity ABA receptor. ABA-induced release of OsNLP4 from the OsSPX4 complex and its subsequent activation of stress-defense genes via N4BS cis-elements are described in Section 3.2.2 (Ma et al., 2025; Wang and Tsay, 2025).

NLP transcription factors constitute the central transcriptional hub through which nitrate and ABA signals are integrated to reprogram growth and stress responses. However, whether AtNRT1.1-derived signals, nitrate binding *per se*, AtNRT1.1-mediated nitrate uptake-coupled Ca²^+^ influx, or both, are strictly necessary or sufficient for NLP activation remains unresolved (Bouguyon et al., 2015; Yanagisawa, 2026). Thus NLPs act as the core nitrate signaling hub, rather than downstream exclusive effectors of AtNRT1.1. Currently, three parallel routes to NLP activation have been identified, each with distinct mechanistic bases and species-specific evidence, namely: transient AtCNGC15-dependent Ca²^+^ spikes downstream of AtNRT1.1 (Wang et al., 2021); autonomous intracellular nitrate sensing by AtNLP7 independent of membrane transceptors (Liu et al., 2022); and LjNRT2.1-mediated NLP signaling in legumes (Misawa et al., 2022). The latter two mechanisms have yet to be confirmed in rice or other non-legume crops. Despite these mechanistic gaps, a transceptor-associated regulatory hub integrating nitrate and stress-related signals to influence NLP-dependent transcription has been reported in selected angiosperm species, although the extent of its molecular conservation across lineages remains to be established (Wang et al., 2018b). To avoid redundancy, the detailed mechanisms of OsNLP4 dual cis-element recognition and the OsNRT1.1B-SPX4-NLP4 signaling cascade are consolidated in Section 3.2.2, while AtNLP7 nitrate sensing and its nuclear retention dynamics are detailed in Section 3.3.1.

#### 4.2.2 Systemic metabolic reprogramming and resource allocation strategies

Following the integration of diverse upstream signals, NRT1.1 ortholog-mediates a global metabolic reprogramming. This process is fundamental for translating environmental assessments at the molecular level into adaptive physiological responses. The core of this reprogramming involves three interconnected layers: the coordination of carbon-nitrogen (C-N) metabolism, the integration of multiple nutrient utilization pathways, and the implementation of context-dependent resource allocation strategies across the whole plant.

A primary function of NRT1.1-mediated signaling is to ensure a balanced supply of metabolic precursors. To meet the high demand for carbon skeletons and reducing power required for nitrogen assimilation, the NLP-centered nitrate signaling axis coactivates key genes in both nitrogen and carbon metabolism, thereby synchronizing their outputs (Durand et al., 2023; Sámano et al., 2024). This coordination is further reinforced at the translational level; for instance, the translation initiation factor *AteIF4E1* enhances AtNRT1.1 translation under low-nitrate conditions, solidifying the C-N metabolic synergy (Li et al., 2023). Beyond C-N balance, NRT1.1 is a central node for integrating the utilization of different mineral nutrients. In rice, nitrate perception by OsNRT1.1B triggers the degradation of the repressor OsSPX4, which concurrently releases transcription factors controlling both nitrogen and phosphorus responses. This coactivation of two major nutrient assimilation pathways ensures systemic nutrient homeostasis and prevents metabolic imbalances caused by the excess of a single nutrient (Hu et al., 2019).

NRT1.1 also governs resource allocation at the whole-plant level, enabling a spectrum of adaptive strategies tailored to specific environmental conditions. Under severe stress, metabolic fluxes are redirected from primary nitrogen assimilation towards the synthesis of protective compounds such as proline and flavonoids, prioritizing survival over growth (Ma et al., 2025). Under moderate stress, mechanisms such as competitive ABA binding allow for a fine-tuned balance between maintaining basal growth and mounting a sufficient defense response. During the reproductive stage, regulatory circuits, such as the OsNR2-OsNRT1.1B module, optimize nitrogen partitioning to the grains, directly influencing yield (Gao et al., 2019; Qi et al., 2023).

The regulatory logic of the NRT1.1 hub holds immense potential for agricultural applications. Genetic evidence demonstrates that enhancing *AtNRT1.1* expression in shoots can improve plant growth and chlorophyll retention under nitrogen-limiting conditions (Sakuraba et al., 2021). Introducing the highly efficient OsNR2-OsNRT1.1B module from *indica* rice into *japonica* varieties has been shown to synergistically enhance nitrogen uptake, assimilation, and translocation to the panicle, resulting in significant improvements in both yield and NUE (Gao et al., 2019). These examples highlight how manipulating the NRT1.1 signaling network can be a powerful strategy for developing more resilient and productive crops.

In conclusion, NRT1.1 orthologs serve as master regulator that translates environmental information into coherent metabolic and physiological action. It establishes a robust metabolic foundation by coordinating C-N metabolism and integrating cross-nutrient pathways. Critically, it directs a flexible resource allocation program that allows plants to navigate a strategic continuum from “growth” to “defense” and “maintenance”. This integration of precise environmental sensing with flexible metabolic adjustments represents a core strategy that enables plants to maximize their fitness and competitiveness in a fluctuating world.

## 5 Physiological outputs and adaptive trade-offs

As a central signaling hub mediating the growth-stress trade-off, the biological significance of NRT1.1 orthologs lie in their capacity to mediate an adaptive balance between survival and growth in fluctuating environments. This section explores how NRT1.1 translates the integration of upstream molecular signals into concrete physiological outputs. By modulating key processes, including root system architecture, ion homeostasis, water use efficiency, and nitrogen metabolism, NRT1.1 mediates a dynamic and hierarchical response that ultimately shapes the overall fitness of the plant.

### 5.1 NRT1.1-mediated remodeling of root architecture: a trade-off between foraging and conservation

The root system architecture (RSA), as the primary interface with the soil, directly reflects a plant’s adaptive state. AtNRT1.1 integrates nutrient and stress signals to modulate the RSA, enabling a flexible switch between an “exploratory foraging” strategy aimed at maximizing resource capture and a “conservative maintenance” strategy designed to conserve energy under adverse conditions.

#### 5.1.1 Phosphorylation-dependent control of lateral root development

The emergence and elongation of lateral roots are pivotal for adaptive root plasticity and are precisely regulated by the phosphorylation status of NRT1.1. Under low-nitrate conditions, *AtNRT1.1* expression is activated by the AtSTOP1-AtTCP20 complex. Subsequent phosphorylation at Thr101 by AtCIPK23 optimizes its membrane localization and function, initiating a cascade that suppresses auxin signaling. This is achieved by downregulating genes involved in auxin biosynthesis (*AtTAR2*), transport (*AtLAX3*), and cell separation (*AtHAE*), culminating in reduced auxin accumulation in lateral root primordia and thereby inhibiting their emergence and preventing futile energy expenditure (Maghiaoui et al., 2020; Zhang et al., 2019b; Tokizawa et al., 2023). Concurrently, phosphorylated NRT1.1 also forms a complex with the kinase AtQSK1 and the plasma membrane H^+^-ATPase AtAHA2, modulating AtAHA2 phosphorylation to inhibit rhizosphere acidification and thus indirectly constrain lateral root growth (Zhu et al., 2024). In stark contrast, under high-nitrate conditions, the predominantly dephosphorylated AtNRT1.1 undergoes clathrin-mediated endocytosis. This process activates an endosome-derived Ca²^+^-ANR1 signaling pathway that stimulates the elongation of existing lateral roots, facilitating efficient resource capture from localized nitrogen-rich patches (Zhang et al., 2019b). This bifunctional, phosphorylation-dependent switch enables plants to dynamically tailor their lateral root development strategy to external nitrate availability.

#### 5.1.2 Adaptive modulation of primary root and root hair growth

To further optimize the resource acquisition interface, AtNRT1.1 concurrently regulates primary root elongation and root hair development. Under low nitrate conditions, AtNRT1.1-mediated Ca²^+^ signaling promotes root hair elongation to expand the nutrient absorption surface (Zhang et al., 2019b). In *Arabidopsis*, this adaptation is further fine-tuned at the translational level, where the initiation factor *AteIF4E1* enhances the expression of *AtNRT1.1* and other root hair-related mRNAs to facilitate acclimation to low-nitrogen environments (Li et al., 2023). Under stress conditions, the RSA is remodeled for enhanced resilience. For example, the rice transcription factor OsSNAC1 activates *OsNRT1.1A/B* expression to promote primary root elongation, a key trait for enhancing drought resistance (Qi et al., 2023).

#### 5.1.3 Shoot-root coordination for whole-plant homeostasis

The NRT1.1-mediated remodeling of the root system is tightly coordinated with shoot development to maintain whole-plant homeostasis. In rice, OsNRT1.1B functions as a nitrate sensor that integrates nitrogen and phosphorus signals through its interaction with OsSPX4. This integration optimizes nitrogen allocation to the shoot, which in turn influences tillering and biomass accumulation, critical for sustaining yield stability in low-nitrogen soils (Hu et al., 2019; Wang et al., 2018b). In *Arabidopsis*, shoot-expressed AtNRT1.1 is induced by low nitrogen and regulates leaf nitrate transport and partitioning. This action sustains chlorophyll content and photosynthetic efficiency, thereby providing metabolic support for root growth and stress adaptation (Sakuraba et al., 2021). Ultimately, NRT1.1 ortholog-mediated nitrogen signals influence the timing of reproductive development by modulating the expression of key flowering genes (e.g., *CONSTANS* [*AtCO*], *FLOWERING LOCUS T* [*AtFT*]) and integrating with hormonal pathways (e.g., auxin, cytokinin), thereby aligning the plant’s life cycle with resource availability to enhance reproductive success (Hao et al., 2025; Ye et al., 2021). This systemic coordination, from root architecture to canopy development and from vegetative growth to reproduction, optimizes resource allocation at the whole-plant level, thereby maximizing overall fitness and adaptability.

### 5.2 Orchestration of ion homeostasis and the rhizosphere microenvironment

Leveraging its dual functionality as both a transporter and a sensor (i.e., a “transceptor”), NRT1.1 orthologs mediate ion balance at cellular and root levels while simultaneously reshaping the chemical microenvironment of the rhizosphere. Through coordinated interactions with other transporters, channels, and transcription factors, NRT1.1 integrates multidimensional signals, including nitrogen status, pH, and heavy metals, to enable precise adaptation in ion uptake, rhizosphere pH regulation, and nutrient co-utilization. This foundational role is critical for plant resilience in heterogeneous soil environments.

#### 5.2.1 Synergistic regulation of ion transport and homeostasis

AtNRT1.1 maintains intracellular and rhizosphere ion homeostasis by forming dynamic regulatory networks. A key mechanism is its H^+^-NO_3_^-^ symport activity, which not only mediates nitrate uptake but also modulates rhizosphere pH. This proton flux indirectly synergizes with K^+^ transporters (e.g., AtAKT1 and AtHAK5) to optimize nitrogen-potassium nutrition (Fang et al., 2020). Furthermore, NRT1.1 participates in protein complexes that fine-tune ion fluxes. For instance, the non-phosphorylated form of AtNRT1.1 (mimicked by AtNRT1.1Thr101A) forms a functional complex with the anion channel SLAH3, creating a feedback loop that reciprocally modulates NO_3_^-^ influx and efflux to prevent excessive rhizosphere acidification.

This pH-dependent regulatory effect also extends to heavy metal ion homeostasis. When both nitrate and ammonium coexist in the rhizosphere, AtNRT1.1-mediated nitrate uptake consumes protons and raises rhizosphere pH, which reduces the solubility and bioavailability of divalent heavy metals such as Pb²^+^ and restricts their entry into root cells. This protective mechanism against lead toxicity is abolished when either nitrogen form is supplied alone, highlighting the essential role of ammonium–nitrate interaction in AtNRT1.1-dependent ion homeostasis (Zhu et al., 2019).

AtNRT1.1 expression and function are also tightly regulated in response to specific environmental stresses. Under acidic conditions, the transcription factor AtSTOP1 directly binds to the *NRT1.1* promoter to activate its expression, which may contribute to H^+^ consumption via H^+^-NO_3_^-^ symport and alleviating acid stress (Ye et al., 2021). In response to ammonium toxicity, AtNRT1.1 acts as a negative regulator of tolerance through a nitrate-independent signaling pathway. At the ion transport level, it upregulates the expression of AtAMT1-type ammonium transporters (particularly AtAMT1;5) which may contribute to increased NH_4_^+^ uptake; at the metabolic level, it suppresses the activities of glutamine synthetase (GS) and glutamate synthase (GOGAT), the core enzymes for ammonium assimilation. The uncoupling of NH_4_^+^ uptake from assimilation leads to free ammonium accumulation in tissues, which further stimulates ethylene biosynthesis and senescence-related gene expression (Jian et al., 2018). The synergy between AtNRT1.1 and SLAH3 also contributes to rhizosphere pH balance under ammonium stress, forming an additional layer of regulatory control (Xiao et al., 2022). In saline environments dominated by ammonium, such as flooded coastal paddy soils, AtNRT1.1 further mediates chloride ion homeostasis disturbance. Ammonium treatment increases AtNRT1.1 protein abundance in roots; in the absence of competitive nitrate substrates, AtNRT1.1 facilitates substantial Cl^-^ influx into root cells, especially in the elongation zone. Excessive chloride accumulation disrupts cellular ion homeostasis and aggravates salt stress damage, and knockout of *AtNRT1.1* significantly reduces root chloride uptake and eliminates ammonium-induced salt hypersensitivity (Liu et al., 2020). Conversely, under iron deficiency, reduced AtNRT1.1 abundance in the shoot apoplast mitigates H^+^ consumption, preventing alkalinization and promoting the release of cell wall-bound iron to counteract chlorosis (Ye et al., 2022). These examples highlight AtNRT1.1’s capacity to make precise adjustments to ion transport by reconfiguring its expression and interaction networks in response to diverse environmental cues. However, changes in transporter expression or protein abundance do not necessarily indicate proportional changes in actual transport capacity. Transport activity may also be influenced by post-transcriptional regulation, protein modification, subcellular localization, substrate availability, and environmental factors such as external pH (Rivero-Marcos and von Wirén, 2026). Therefore, the effects described above should be interpreted as changes in regulatory potential unless directly supported by measurements of ion flux or uptake capacity.

This regulatory logic is conserved across species but adapted to specific ecological niches. The phosphorylation of AtNRT1.1 at Thr101 in *Arabidopsis* dynamically tunes its NO_3_^-^ transport affinity, with the phosphomimetic Thr101D variant exhibiting high-affinity transport under low nitrate (Bouguyon et al., 2015). In rice, the vacuolar-localized OsNRT1.1A functions as an ammonium-inducible transporter, coordinating NO_3_^-^ and NH_4_^+^ utilization to adapt to flooded paddy conditions (Wang et al., 2018b). This pattern of a conserved regulatory core with species-specific adaptations underscores the evolutionary significance of NRT1.1 orthologs as central hub for soil environments.

Collectively, these findings illustrate that NRT1.1 orthologs serve as a nexus integrating nitrate, ammonium, and stress signals. Its functional output depends critically on the prevailing nitrogen form and the specific stress context, and it coordinates rhizosphere ion balance, intracellular transport and metabolic homeostasis through multi-level regulatory networks.

#### 5.2.2 Rhizosphere engineering: linking nutrient bioavailability and microbial interactions

The NRT1.1 ortholog-mediated modulation of proton fluxes transcends simple ion transport, actively engineering the rhizosphere to influence nutrient availability and shape microbial communities. The resulting pH alterations directly impact the solubility and bioavailability of essential nutrients such as phosphorus and iron (Ye et al., 2021, 2022). Moreover, this chemical conditioning of the soil, coupled with changes in root exudation profiles influenced by NRT1.1 activity, exerts selective pressure on the root-associated microbiome.

An example is found in rice, where natural variations in *OsNRT1.1B* are associated with the enrichment of bacteria capable of organic nitrogen mineralization. This establishes a beneficial feedback loop: the host genotype shapes a microbial community that, in turn, enhances nitrogen cycling efficiency (Zhang et al., 2019a). This highlights a sophisticated regulatory axis: host genotype - microbial community - nutrient availability. The interaction mechanisms exhibit species-specific adaptations: in the terrestrial plant *Arabidopsis*, AtNRT1.1-AtSTOP1-AtSLAH3 interactions prioritize immediate chemical optimization of the rhizosphere (Jian et al., 2018; Xiao et al., 2022). In contrast, rice employs a multi-faceted strategy suited to paddy ecosystems, where vacuolar OsNRT1.1A buffers intracellular NH_4_^+^/NO_3_^-^, and plasma membrane-localized OsNRT1.1B integrates nitrate-phosphate signals to modulate both rhizosphere pH and the beneficial microbiome (Wang et al., 2018b; Hu et al., 2019). Fundamentally, NRT1.1 orthologs convert proton transport into a powerful tool for systemic adaptation, coordinating the soil chemical environment, nutrient networks, and microbial ecology.

#### 5.2.3 The nitrogen-phosphorus trade-off: a paradigm of nutrient co-regulation

NRT1.1 family proteins occupy a central position in mediating the dynamic trade-off between N and P acquisition. This coordination enables plants to align their nutrient uptake capacity with the most limiting element, avoiding toxic ion accumulation and wasteful energy expenditure. Notably, this crosstalk is implemented through evolutionarily divergent, species-specific regulatory modules.

In *Arabidopsis*, the N-P trade-off is governed by a hierarchical transcriptional network centered on the NIGT1 subfamily, which serves as a convergent node for both N and P signaling (Maeda et al., 2018; Wang et al., 2020b; Ohama and Yanagisawa, 2024). Under nitrate-replete conditions,AtNRT1.1-mediated Ca²^+^-CPK signaling activates NLP transcription factors such as AtNLP7, which directly bind to nitrate-responsive elements in *AtNIGT1* promoters and induce their expression. Accumulated AtNIGT1 proteins function as transcriptional repressors that suppress a suite of nitrate transporter genes, including both the high-affinity transporter *AtNRT2.1* and the dual-affinity transceptor *AtNRT1.1*, to prevent excessive N uptake. Concurrently, NIGT1 proteins repress the expression of *AtSPX* genes (e.g., *AtSPX1*, *AtSPX2*, *AtSPX4*). Since AtSPX proteins act as negative regulators that sequester the P starvation master regulator AtPHR1, AtNIGT1-mediated *AtSPX* repression de-represses *AtPHR1* activity and primes the plant for P deficiency responses (Kiba et al., 2018).

Conversely, under P starvation, activated AtPHR1 directly binds to AtP1BS motifs in *AtNIGT1* promoters (which partially overlap with AtNIGT1-binding sites) and strongly upregulates *AtNIGT1* expression. The elevated AtNIGT1 repressors in turn directly target the *AtNRT1.1* promoter and downregulate its transcription, restricting nitrate uptake to match the reduced metabolic demand under P limitation (Maeda et al., 2018; Ueda et al., 2020). This AtPHR1–AtNIGT1–AtNRT1.1 transcriptional cascade forms a negative feedback loop that fine-tunes N acquisition in response to P status.

Rice employs an evolutionarily divergent post-translational module anchored at the plasma membrane transceptor OsNRT1.1B. Nitrate perception promotes the recruitment of the E3 ligase OsNBIP1, which ubiquitinates and triggers the degradation of the cytosolic repressor OsSPX4 (Hu et al., 2019). Degradation of OsSPX4 simultaneously releases two sequestered transcription factors: OsNLP3/OsNLP4 (rice NIN-LIKE PROTEIN nitrate signaling transcription factors; OsNLP3 primarily drives vegetative nitrogen assimilation, while the *indica* allele of OsNLP4 contains three coding-region SNPs that enhance NRE-binding affinity to activate nitrate assimilation genes including OsNIA1 and OsNiR1 (Wu et al., 2021, 2025; Xu et al., 2026), and OsPHR2 (Wu et al., 2021) to upregulate phosphate transporters (Hu et al., 2019).

Collectively, these evolutionarily divergent regulatory modules converge on NRT1.1 orthologs as the primary nitrate sensor. Whether through the AtNIGT1-mediated transcriptional network in *Arabidopsis* or the OsSPX4-dependent protein interaction module in rice, NRT1.1 functions as the core decision point that balances growth-promoting nutrient acquisition and starvation stress adaptation.

### 5.3 Coordination of nitrogen metabolism: the nexus of signaling and phenotype

Nitrogen metabolism represents a core physiological output of NRT1.1 ortholog activity and serves as the critical interface connecting upstream signaling with downstream phenotypic plasticity. By regulating nitrate uptake, assimilation, and metabolic partitioning, NRT1.1 establishes a regulatory circuit of “nutrient signal - metabolic reprogramming - stress adaptation”, thereby providing the metabolic foundation for the growth-stress trade-off.

#### 5.3.1 Coupling of nitrate uptake and assimilation

AtNRT1.1 is tightly coupled with downstream nitrogen assimilation pathways, ensuring a dynamic balance between nitrate acquisition and its subsequent metabolic conversion. In rice, an elite allele of the nitrate reductase gene *OsNR2* (prevalent in *indica* varieties) synergizes with OsNRT1.1B through a feedforward loop. This interaction not only promotes nitrate uptake but also directly enhances the activity of assimilatory enzymes, significantly improving NUE and grain yield (Gao et al., 2019). At the subcellular level, the tonoplast-localized OsNRT1.1A regulates the storage and remobilization of vacuolar nitrate. It upregulates the expression of assimilation genes (e.g., *OsNIA1*, *OsNiR1*) and, under nitrogen deficiency, promotes the nuclear localization of the transcription factors OsNLP3/4, thus orchestrating nitrogen storage, signaling, and assimilation (Wang et al., 2018a). Furthermore, under conditions such as ammonium toxicity, the functional coupling between AtNRT1.1 and the anion channel AtSLAH3 is essential. Their coordinated regulation of NO_3_^-^ fluxes help mitigate rhizosphere acidification while supplying crucial carbon skeletons (e.g., α-ketoglutarate) required for ammonium assimilation via the GS/GOGAT cycle, thereby maintaining nitrogen metabolic homeostasis (Jian et al., 2018; Xiao et al., 2022).

#### 5.3.2 Metabolic reprogramming for stress resilience

In response to environmental stress, the NRT1.1-centered nitrogen network is strategically reprogrammed, redirecting limited nitrogen resources toward pathways essential for survival. Under drought, rice OsNRT1.1B functions as an ABA receptor, and its signaling output shifts nitrogen flux toward the synthesis of osmolytes such as proline. Through the OsSPX4-OsNLP4 module, it integrates nitrogen and phosphorus signals to ensure that nitrogen is prioritized for maintaining cellular turgor and antioxidant capacity rather than for vegetative growth (Ma et al., 2025). This metabolic reallocation extends to other nutrient stresses. For example, the downregulation of shoot AtNRT1.1 under iron deficiency is an adaptive strategy that facilitates the remobilization of cell wall-bound iron by modulating apoplastic pH, demonstrating a direct link between nitrate signaling and iron homeostasis (Ye et al., 2022). Similarly, by inhibiting auxin-dependent lateral root emergence under low nitrogen, AtNRT1.1 effectively reallocates carbon and nitrogen from root branching toward primary root elongation and the synthesis of storage compounds, a crucial strategy for nutrient-scarce conditions (Zhang et al., 2019b). During reproductive development, NRT1.1-related pathways (e.g., OsSNAC1-OsNRT1.1 and OsNR2-OsNRT1.1B) fine-tune nitrogen translocation to the grains. This process is critical for safeguarding reproductive output under stress, exemplifying the ultimate trade-off between somatic maintenance and reproductive investment (Gao et al., 2019; Qi et al., 2023).

In essence, the physiological outputs mediated by NRT1.1 orthologs, from shaping root architecture and engineering the rhizosphere to partitioning nitrogen metabolites, collectively represent a sophisticated strategy for plant environmental adaptation. NRT1.1 orthologs acts as a master regulator that translates disparate environmental cues into a unified decision-making framework, continuously optimizing the allocation of finite resources between growth and defense across all organizational scales. The physiological outputs of NRT1.1 orthologs under different environmental cues are summarized in **Table 2**.

**Table 2:** Comparative physiological outputs of NRT1.1 under different environmental cues.

Environmental cue	Species	NRT1.1 status/mechanism	Ion/pH homeostasis	Root remodeling	Water use/stomata	N metabolism	Overall adaptive strategy	Reference
Low nitrate	*Arabidopsis thaliana*; *Oryza sativa*	Thr101 phosphorylation (CIPK23)*^At^*; high-affinity mode *^At^*,*^Os^*	H^+^-NO_3_^-^ symport; rhizosphere pH increase; synergistic K^+^ uptake*^At^*	Lateral root suppression; primary root/root hair elongation *^At^*,*^Os^*	Stomatal closure promoted (T101D mutant phenotype)	NR/NiR activation; C-N coordination *^At^*,*^Os^*	Conservative foraging	Zhang et al., 2019b; Tokizawa et al., 2023; Maghiaoui et al., 2020; Kou et al., 2025
High nitrate	*Arabidopsis thaliana*; *Oryza sativa*	Thr101 dephosphorylated; low-affinity mode*^At^*,*^Os^*;nitrate-responsive OsNRT1.1B signaling*^Os^*	H^+^-NO_3_^-^ symport; rhizosphere acidification*^At^*	Lateral root elongation (Ca²^+^-ANR1 pathway)*^At^*	Not directly specifiedt	SPX4 degradation →OsNLP3/AtNLP7 and OsPHR2 → N and P assimilation genes *^At^*,*^Os^*	Proactive nutrient exploitation	Zhang et al., 2019b; Hu et al., 2019
Drought/ABA	*Arabidopsis thaliana*; *Oryza sativa*	ABA binds Arg48*^Os^*; SnRK2 phosphorylates Ser585 *^At^*	Indirect maintenance of K^+^/Na^+^ maintenance *^At^*,*^Os^*	Primary root elongation (OsSNAC1-OsNRT1.1A/B)*^Os^*	Stomatal closure; increased water use efficiency *^At^*,*^Os^*	Proline synthesis prioritized; N assimilation suppressed*^Os^*	Survival priority; water conservation	Ma et al., 2025; Su et al., 2021; Qi et al., 2023
Ammonium toxicity/acidic stress	*Arabidopsis thaliana*	NRT1.1 – SLAH3 complex *^At^*; STOP1-TCP20 activation *^At^*	H^+^-NO_3_^-^ consumes protons → alleviates rhizosphere acidification; GS/GOGAT for NH_4_^+^ *^At^*	Lateral root inhibition (acidic stress)	Not directly reported	Organic acid fuels GS/GOGAT; NH_4_^+^ reassimilation *^At^*	pH homeostasis; toxicity mitigation	Jian et al., 2018; Xiao et al., 2022; Tokizawa et al., 2023
Phosphate starvation	*Arabidopsis thaliana*; *Oryza sativa*	Transcriptional repression (NIGT1.2, activated by PHR1)*^At^*; SPX4 protein stabilization *^Os^*	Prioritize P over N uptake; reduced H^+^-NO_3_^-^ symport *^At^*,*^Os^*	Not directly reported	Not reported	NIGT1.2 represses NRT1.1; NLP7 antagonizes*^At^*; SPX4 sequesters NLPs *^Os^*	Nutrient prioritization (P over N)	Maeda et al., 2018; Ueda et al., 2020; Hu et al., 2019
Iron deficiency	*Arabidopsis thaliana*	Reduced NRT1.1 protein in shoot apoplast*^At^*	Reduced H^+^ consumption → prevents alkalinization →release of cell wall Fe*^At^*	Not directly reported	Not reported	Not directly reported	Iron remobilization; chlorosis mitigation	Ye et al., 2022

The species listed in the “species” column indicate the plant species in which the reported function was experimentally investigated. At, *Arabidopsis thaliana*; Os, *Oryza sativa*. The presence of an NRT1.1 ortholog in other plant species does not by itself establish conservation of the corresponding function. “Not directly reported” indicates that the corresponding output was not assessed in the cited study.

## 6 Concluding remarks and future perspectives

This review identifies NRT1.1 as a key signaling hub linking nitrate availability with stress responses and provides a framework for understanding the growth–stress trade-off in plants. However, the mechanistic evidence supporting this framework remains concentrated in *Arabidopsis* and a limited number of crop species; its conservation and coordinated operation in other crops require further experimental validation. Future work should test NRT1.1-associated regulatory relationships under combined stresses and field-relevant conditions. Integrating natural genetic variation, functional validation, and precision breeding may help assess whether these pathways can contribute to improved nitrogen-use efficiency and stress resilience. Such applications will require careful consideration of species-specific functions, environmental context, and the actual effects of candidate alleles on plant performance.
