# Supplementary material for: NRT1.1 as central signaling hub orchestrating plant growth and stress trade-offs
Source: Front Plant Sci. 2026 Sep 23;17:1965737. doi: 10.3389/fpls.2026.1965737 (PMC13643022; doi:10.3389/fpls.2026.1965737)
Supplement: Supplementary file 1 [file Table1.docx]

Table S1 Glossary of core gene abbreviations, full gene names, encoded protein functions, and associated references

| Gene Abbreviation | Full Gene Name | Encoded Core Protein Function | Reference |
| --- | --- | --- | --- |
| AtNRT1.1 (CHL1/NPF6.3) | *Arabidopsis thaliana* NITRATE TRANSPORTER 1.1 / CHLORATE RESISTANT 1 / NPF6.3 | Dual-affinity nitrate transceptor with nitrate sensing function; **s**ubject to AtSnRK2-mediated inhibitory phosphorylation at Ser585 under ABA stress; contains conserved Thr101 switch | Tsay et al., 1993; Su et al., 2021 |
| OsNRT1.1A/B/C | *Oryza sativa* NRT1.1 paralogs A/B/C | OsNRT1.1B: plasma membrane dual transceptor with high-affinity ABA-binding Arg48 site (rice-specific); OsNRT1.1A: vacuolar nitrate storage transporter; OsNRT1.1C: tissue-restricted low-expression paralog | Hu et al., 2015; Ma et al., 2025 |
| ZmNRT1.1B | *Zea mays* NRT1.1B | Maize functional ortholog of NRT1.1 regulating nitrate uptake and crop nitrogen use efficiency | Cao et al., 2024 |
| AtNRT2.1/AtNRT2.2 | *Arabidopsis thaliana* NITRATE TRANSPORTER 2.1/2.2 | Major high-affinity nitrate transporters; AtNRT2.1 participates in independent nitrate-NLP signaling cascade | Li et al., 2007; Misawa et al., 2022 |
| LjNRT2.1 | *Lotus japonicus* NRT2.1 | Legume nitrate transporter controlling NLP-dependent nodulation signaling | Misawa et al., 2022 |
| AtNLP7 | *Arabidopsis thaliana* NIN-LIKE PROTEIN 7 | Master nitrate-responsive transcription factor governing primary nitrate response (PNR) | Maeda et al., 2018; Liu et al., 2017 |
| AtNLP2 | *Arabidopsis thaliana* NIN-LIKE PROTEIN 2 | Early nitrate TF linking nitrate assimilation with carbon metabolism | Konishi et al., 2021 |
| OsNLP3 / OsNLP4 | *Oryza sativa* NIN-LIKE PROTEIN 3 / 4 | Rice nitrate transcription factors; OsNLP3 mediates basal vegetative growth; indica OsNLP4 allele enhances NRE binding and NUE; OsNLP4 also activates ABA-responsive genes via N4BS motifs | Hu et al., 2019; Wu et al., 2025 |
| AtNIGT1.1–1.4 | *Arabidopsis* NITRATE-INDUCIBLE GARP repressors | GARP-type transcriptional repressors inhibiting *NRT1.1* and SPX family genes to balance N-P signaling | Kiba et al., 2018; Maeda et al., 2018 |
| AtPHR1 / OsPHR2 | PHOSPHATE STARVATION RESPONSE 1 / 2 | Myb-type master transcription factors controlling phosphate starvation transcriptional programs | Maeda et al., 2018; Hu et al., 2019 |
| AtSPX1/2/4 / OsSPX4 | SYG1/Pho81/XPR1 domain-containing proteins | Cytoplasmic repressors sequestering PHR1 and NLP paralogs under low nitrate | Ueda et al., 2020; Hu et al., 2019 |
| OsNR2 (OsNIA2) | *Oryza sativa* Nitrate Reductase 2 | High-activity NADH/NADPH nitrate reductase; forms positive feedback loop with *OsNRT1.1B* to boost rice NUE | Gao et al., 2019 |
| OsNIA1 | *Oryza sativa* Nitrate Reductase 1 | NADH-specific nitrate reductase for basal nitrate assimilation and NO synthesis under low temperature | Xu et al., 2026 |
| OsNiR1 | *Oryza sativa* Nitrite Reductase 1 | Plastidic ferredoxin-dependent nitrite reductase core of nitrate assimilation pathway | Wu et al., 2021 |
| OsSNAC1 | *Oryza sativa* Stress-Responsive NAC 1 | Salt-stress NAC TF activating *OsNRT1.1A/B* to remodel root architecture | Qi et al., 2023 |
| AtSTOP1 | Sensitive to Proton Toxicity 1 | C2H2 zinc-finger TF coordinating acid stress response and activating *NRT1.1* | Tokizawa et al., 2023 |
| AtTCP20 | Teosinte Branched1/Cycloidea/PCF 20 | Class-I TCP co-factor forming STOP1-TCP20 complex to regulate *NRT1.1* transcription | Tokizawa et al., 2023 |
| AtCBL1/9 | Calcineurin B-LIKE PROTEIN 1/9 | Calcium sensor proteins activating CIPK23 to phosphorylate NRT1.1 Thr101 | Ho et al., 2009 |
| AtCIPK8 / AtCIPK23 | CBL-INTERACTING PROTEIN KINASE 8/23 | CIPK23: low-nitrate kinase activating NRT1.1 phosphorylation; CIPK8: high-nitrate negative feedback inhibitor | Rashid et al., 2020 |
| AtCPK6/10/30/32 | CALCIUM-DEPENDENT PROTEIN KINASE 6/10/30/32 | CPK10/30/32 promote NLP nuclear retention; CPK6 phosphorylates NRT1.1 to suppress nitrate transport under stress | Liu et al., 2017; Ma et al., 2023 |
| AtSnRK2.6 (OST1) | SUCROSE NON-FERMENTING 1-RELATED KINASE 2.6 / OPEN STOMATA 1 | ABA-activated kinase targeting AtNRT1.1 Ser585 to inhibit nitrate uptake | Su et al., 2021 |
| AtCNGC15 | *Arabidopsis thaliana* CYCLIC NUCLEOTIDE-GATED CHANNEL 15 | Plasma membrane channel interacting with AtNRT1.1 to generate transient nitrate-triggered Ca²⁺ spike | Wang et al., 2021 |
| OsCNGC14/16 | *Oryza sativa* CYCLIC NUCLEOTIDE-GATED CHANNEL 14/16 | Rice homologs mediating OsNRT1.1B-dependent Ca²⁺ influx | Wang et al., 2025 |
| AtNBIP1 | NRT1.1B-INTERACTING PROTEIN 1 | Rice E3 ubiquitin ligase triggering OsSPX4 degradation under high nitrate | Hu et al., 2019 |
| AtSLAH3 | SLOW ANION CHANNEL-ASSOCIATED 3 | Anion channel coordinating NRT1.1 to balance rhizosphere pH and nitrate efflux | Xiao et al., 2022 |
| AtAKT1 / AtHAK5 | ARABIDOPSIS K⁺ TRANSPORTER 1 / HIGH-AFFINITY K⁺ TRANSPORTER 5 | Potassium transporters cooperating with NRT1.1 for N-K ion homeostasis | Fang et al., 2020 |
| AtAMT1;5 | AMMONIUM TRANSPORTER 1;5 | High-affinity ammonium transporter induced during ammonium toxicity stress | Jian et al., 2018 |
| AtPIN2 | PIN-FORMED 2 | Auxin efflux transporter modulated by NRT1.1 to control lateral root outgrowth | Bouguyon et al., 2016 |
| AtLAX3 | LIKE AUXIN RESISTANT 3 | Auxin influx carrier regulating lateral root primordia development | Maghiaoui et al., 2020 |
| AtTAR2 | TRYPTOPHAN AMINOTRANSFERASE RELATED 2 | Auxin biosynthesis gene repressed under low nitrate | Zhang et al., 2019 |
| AtAFB3 | AUXIN SIGNALING F-BOX 3 | Auxin receptor induced under high nitrate to stimulate lateral root elongation | Vidal et al., 2010 |
| AtANR1 | ARABIDOPSIS NITRATE REGULATED 1 | Nitrate TF controlling lateral root elongation downstream of endosomal NRT1.1 signaling | Zhang et al., 2019 |
| AtQSK1 | QUASIMODO-LIKE SERINE KINASE 1 | Kinase complexed with NRT1.1 and AHA2 to modulate rhizosphere acidification | Zhu et al., 2024 |
| AtAHA2 | ARABIDOPSIS H⁺-ATPASE 2 | Plasma membrane proton pump co-regulating root zone pH with NRT1.1 | Zhu et al., 2024 |
